# Microbial Communities in Ferromanganese Sediments from the Northern Basin of Lake Baikal (Russia)

**DOI:** 10.3390/microorganisms11071865

**Published:** 2023-07-24

**Authors:** Anna Lomakina, Sergei Bukin, Olga Shubenkova, Tatyana Pogodaeva, Vyacheslav Ivanov, Yuri Bukin, Tamara Zemskaya

**Affiliations:** Limnological Institute, Siberian Branch of the Russian Academy of Sciences (LIN SB RAS), 664033 Irkutsk, Russiatzema@lin.irk.ru (T.Z.)

**Keywords:** microbial communities, Lake Baikal, Fe-Mn layers, diversity of 16S rRNA sequences, metagenome-assembled genomes (MAGs)

## Abstract

We analyzed the amplicons of the 16S rRNA genes and assembled metagenome-assembled genomes (MAGs) of the enrichment culture from the Fe-Mn layer to have an insight into the diversity and metabolic potential of microbial communities from sediments of two sites in the northern basin of Lake Baikal. Organotrophic *Chloroflexota*, *Actionobacteriota*, and *Acidobacteriota*, as well as aerobic and anaerobic participants of the methane cycle (*Methylococcales* and *Methylomirabilota*, respectively), dominated the communities of the surface layers. With depth, one of the cores showed a decrease in the proportion of the *Chloroflexota* and *Acidobacteriota* members and a substantial increase in the sequences of the phylum Firmicutes. The proportion of the *Desulfobacteriota* and *Thermodesulfovibronia* (*Nitrospirota*) increased in another core. The composition of archaeal communities was similar between the investigated sites and differed in depth. Members of ammonia-oxidizing archaea (*Nitrososphaeria*) predominated in the surface sediments, with an increase in anaerobic methanotrophs (*Methanoperedenaceae*) and organoheterotrophs (*Bathyarchaeia*) in deep sediments. Among the 37 MAGs, *Gammaproteobacteria*, *Desulfobacteriota*, and *Methylomirabilota* were the most common in the microbial community. Metagenome sequencing revealed the assembled genomes genes for N, S, and CH_4_ metabolism for carbon fixation, and genes encoding Fe and Mn pathways, indicating the likely coexistence of the biogeochemical cycle of various elements and creating certain conditions for the development of taxonomically and functionally diverse microbial communities.

## 1. Introduction

Crusts, nodules, and micronodules formed by iron oxides and manganese oxides with various geochemical and mineral compositions are found in numerous lakes [1], including Lake Vermilion, Minnesota [2], Lake Charlotte, Nova Scotia [3], Ship Harbour Lake, Nova Scotia and Mosque Lake, Ontario [1], Lake Superior and Lake Michigan, North America [4,5], Lake Constance (Bodensee), Central and Western Europe [6], Lake Baikal, and Lake Onega, Russia [7,8,9,10,11]. The elemental and isotopic composition of sediments and nodules in these lakes reflected environmental conditions and the importance of anthropogenic and geological sources in the formation of Fe-Mn layers and nodules. The diagenetic formations of Fe-Mn nodules and crusts show elevated concentrations of Cu, Ni, Zn, Li, Mo, and Cd, while hydrothermal ones normally show low concentrations of secondary metals [12]. Shallow-water Fe-Mn formations do not accumulate rare elements, are enriched in iron relative to manganese, and have high concentrations of detritus, e.g., [13,14]. 

Different research teams have studied the formation, composition, typification, and origin of Fe-Mn in Lake Baikal, the world’s oldest and deepest lake [9,11,15,16,17,18,19]. Obvious zones of Fe and Mn accumulation in the form of interlayers or crusts were detected in the sediments of all Baikal basins (Russia), as well as on the underwater Akademichesky (Academic) Ridge [8]. They can be broadly subdivided into an upper accumulation at the O_2_/Mn(II) redox boundary and one or more layers buried within the reducing part of the sediment. In terms of genesis, Fe-Mn layers are predominantly classified as diagenetic [16]; although the participation of hydrothermal processes in the formation of Fe-Mn crusts was also suggested, in particular near the Ushkany Islands [16,20]. In Lake Baikal the dynamics of Fe-Mn layers explain the past climate changes [8,21] or tectonic events and subsequent redistribution of Fe and Mn [11]. The study of Fe-Mn crusts and layers in different basins of Lake Baikal revealed two types of diagenetic redistribution [8,9]. One type was characterized by the sediments of the southern and central basins, where they are poorly accumulated near the surface of sediments due to the intensive mineralization of the buried organic matter. Another type was detected in the northern basin, where massive interlayers enriched in iron and manganese are rather formed in the upper oxidized sedimentary layer, near the redox boundary. The role of hydrothermal processes in the diagenesis of Fe-Mn layers and crusts near the Ushkany Islands has so far remained unknown [22]. 

Some studies have indicated that the formation of Fe-Mn crusts involves abiotic and microbiological processes (biogenesis) [23,24]. Biogenesis in the formation of Fe-Mn crusts has not been fully described, although the presence of microorganisms in them is a proven fact, but the data on the intra- and mutual variations of microbial communities in the crusts are limited [25,26,27]. Morphologically diverse bacteria catalyze the precipitation of Fe and Mn oxides [2,28,29], and bacterial deposition occurs 60 times faster than abiotic processes in the comparable environmental conditions [3,30]. Microorganisms reducing Fe(III) and Mn(IV) can oxidize a wide range of organic compounds often completely to carbon dioxide [11,31]. Iron(III)- and sulfate reduction are the most important electron sources of both in freshwater and marine anoxic environments, e.g., [32]. The reduction of Fe(III) can be mediated by sulfide [33], organic compounds, hydrogen, elemental sulfur (e.g., [34,35]), or nitrate [36]. The ability to use metal ions of two different oxidation states was also proved for archaea that use them as final electron acceptors for anaerobic respiration [37,38]. Archaea can use iron (ferrihydrite) and manganese (birnessite) as electron acceptors (in addition to sulfate, nitrate ions) during anaerobic oxidation of methane (AOM) [39,40,41]. These are members of the family *Methanoperedenaceae* (formerly ANME-2d), *Candidatus* Methanoperedens ferrireducen among them, that use Fe(III) as the final electron acceptor for AOM [40]. Two other members of this family, Ca. Methanoperedens manganicus and Ca. Methanoperedens manganireducens, use Mn(IV) oxides as electron acceptors during AOM [42]. The archaea, *Geoglobus acetivorans* and *Geoglobus ahangari*, can also use Fe(III) as a final electron acceptor [43].

Och et al. [9] suggested the mechanism for the formation of ferromanganese layers of the diagenetic genesis in Lake Baikal: dynamic growth of Fe and Mn in oxidized layers directly below the layer of maximum O_2_ penetration, slower reductive dissolution with subsequent burial of Fe-Mn oxides, and further initiation of the formation of new dynamic Fe-Mn layers above. According to the authors, the dissolution of the buried Fe-Mn oxide layer is ultimately controlled by AOM using SO_4_^2−^ and/or Fe(III) as electron acceptors in deeper sediments, with the formation of an upper dynamic Fe-Mn oxide layer due to the O_2_ diffusion from the water column in the sediments [9,11]. The hypothesis of the diagenetic formation of Fe-Mn layers resulting from the biogenic activity in the freshwater sediments of Lake Baikal corresponds to the studies by Dubinina [44], Granina [8], and Zakharova et al. [45], as well as to our previous results of the study of communities in the sediments with Fe-Mn layers near Bolshoy Ushkany Island [22]. To develop this hypothesis, we investigated the diversity and structure of microbial communities in the sediments of the northern basin of Lake Baikal, where Granina [8] and Och et al. [9] indicated a different type of diagenetic redistribution of Fe and Mn. 

This article discusses the results of a study of the physicochemical characteristics, structure, and diversity of microbial communities in the sediments of the northern basin of Lake Baikal, with Fe-Mn layers based on the analysis of 16S rRNA gene amplicons and genomes from MAGs, as well as the potential role of Fe-Mn layers in biogenesis. 

## 2. Materials and Methods 

### 2.1. Description of Sampling Sites, Sample Collection, and Pretreatment

**Figure 1:**
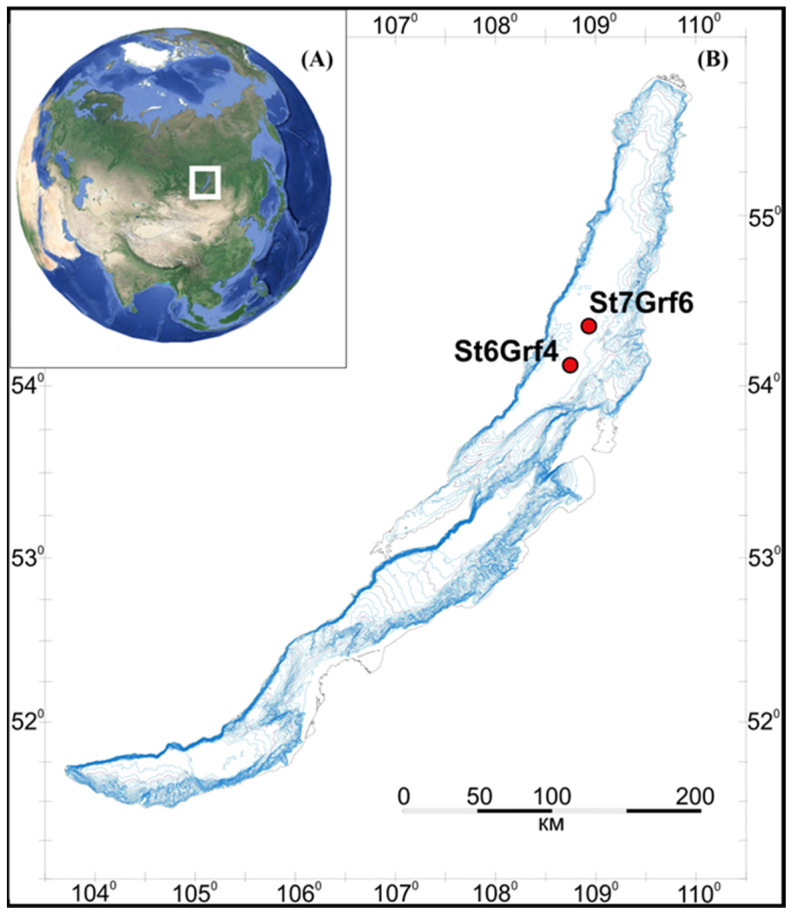
Geographic location of Lake Baikal (**A**) and the location of the sediments sampling area (**B**).

Sediment samples were collected during the 2020 expedition aboard the research vessel (RV) “G.Yu. Vereshchagin” using NIOZ-type box core (Grf), which was rectangular in shape, 0.78 m^−2^, and 70 cm high from the site located in the northern basin of Lake Baikal (Figure 1A). In the course of the investigation, we collected sediment samples from two sites (St6Grf4 and St7Grf6, Figure 1B) with Fe-Mn layers. Chemical analysis of pore waters and lithology was carried out in the laboratory onboard the vessel. The sediments sampled for DNA extraction were stored in liquid nitrogen until laboratory analysis was performed. For the chemical analysis and molecular investigations, the surface layer 0–5 cm, 10–11 cm, 12–13 cm (Fe-Mn layer), 22–23 cm, 23–24 cm (Fe-Mn layer), 24–25 cm, and 29–30 cm were taken from St6Grf4; sediment samples 0–5 cm, 6–7 cm (Fe-Mn layer), 15–16 cm, 19–20 cm, 20–21 cm (Fe-Mn layer), and 25–26 cm were taken from St7Grf6.

### 2.2. Chemical Analysis

Pore water extractions were obtained from samples (100 g each) on board by sequential centrifuging the sample for 20 min at 5000× *g* and then for 10 min at 12,000× *g*. Chemical analysis was carried out as described previously [46]. The pore water anion and cation contents were determined at the Collective Instrumental Center “Ultramicroanalysis” of LIN SB RAS. Pore water anion (SO_4_^2−^, NO_3_^−^, HCO_3_^−^, and CH_3_COO^−^) concentrations were measured on board by liquid chromatography using Milikhrom-A-02 (Novosibirsk, Russia) immediately after preparation (relative error 5–10%) [47]. Cation (Na^+^, K^+^, Ca^2+^, and Mg^2+^, Mn^2+^, and Fe^2+^) concentrations were determined using atomic absorption and flame emissivity methods (relative error 3–5%). NH_4_^+^ was determined on board by colorimetric methods (relative error 5%). Measurements of pH and Eh were carried out in sediments using pH 3310 (“WTW”, Weilheim, Germany).

### 2.3. Methane Concentration

Dissolved methane concentrations in the sediments were determined using headspace analysis [48] on an EKHO-PID chromatograph (Russia) (flame ionization detector, 2 m packed column with an inner diameter of 2 mm; the Porapak Q sorbent, isothermal mode, column temperature 100 °C, injector temperature 100 °C, and detector 150 °C). Gas volume was 0.5 mL. The error of the determination method was 7%.

### 2.4. DNA Isolation, PCR, and Sequencing of 16S rRNA Genes 

Sediments for DNA extraction were sampled immediately after collection: 10 g of sediments were packed in sterile foil and placed in liquid nitrogen until laboratory analysis. DNA was extracted from sediments by enzymatic lysis, followed by phenol-chloroform extraction [49]. For PCR amplification of 16S rRNA gene fragments of bacteria, including the variable region V2–V3, the universal primers, 16S_BV2f (5′-AGTGGCGGACGGGTGAGTAA-3′) and 16S_BV3r (5′-CCGCGGCTGCTGGCAC-3′) [50], as well as the following procedure, were used: 95 °C for 15 min; 96 °C for 30 s; 53 °C for 60 s; 72 °C for 40 s (25 cycles), and 72 °C for 10 min. For PCR amplification of 16S rRNA gene fragments of archaea, the primers that included the variable region V5–V6, Arch-0787f (5′-ATTAGATACCCSBGTAGTCC-3′), and Arch-1059r (5′-GCCATGCACCWCCTCT-3′) [51], as well as the following procedure, were used: 95 °C for 15 min; 96 °C for 30 s, 50 °C for 60 s, 72 °C for 40 s (25 cycles), and 72 °C for 10 min. The libraries were analyzed using Illumina MiSeq Standard Kit v.3 (Illumina) at the Core Centrum “Genomic Technologies, Proteomics and Cell Biology” in ARRIAM.

### 2.5. 16S rRNA Sequence Processing and Statistical Analysis

The resulting forward and reverse raw paired-end sequencing reads were trimmed from the end to 270 bp using Trimmomatic version 0.39 [52] to remove positions in which more than 25% of the reads had bases with relative quality scores < 20, as calculated using FastQC v. 0.11.9 [53]. Further analyses were conducted using R v4.2.2 in RStudio v2022.12.0. The libraries were processed using DADA2 v1.26 [54], following the suggested workflow [55]. Reads were filtered and merged based on a minimum overlap of 12 bp. Chimeras and amplicon sequence variants (ASVs) that were observed in only one sample were filtered out. The representative sequences were taxonomically classified against SILVA 16S rRNA gene reference database release 138_1 (http://www.arb-silva.de, accessed on 31 January 2023). The ASVs that were taxonomically unclassified at phylum rank or were not assigned to bacterial or archaeal lineages, as well as ASVs that were taxonomically assigned to mitochondria and chloroplast, were excluded from further analysis. The 16S rRNA sequences were deposited in the NCBI Sequence Read Archive under Bioproject PRJNA875570 and IDs SAMN35823513 for St6Grf4 and SAMN35823513 for St7Grf6.

Alpha diversity values (ACE, Chao1 indices, Shannon’s and Inverse Simpson’s diversity) were calculated for non-normalized sequence data using phyloseq v1.34.0 [56]. For other statistical analysis, all data on the concentration of ions in pore waters, as well as ASV relative abundance, were normalized by unitization with a zero minimum [(x-min)/range]. 

Comparisons of environmental parameters and beta diversity of microbial communities using non-metric multidimensional scaling (NMDS), as well as statistical tests and visualization, were performed with the vegan [57], stats, and ggplot2 [58] packages.

### 2.6. Preparation of Enrichment Culture from the Sedimentary Layer Enriched in Fe and Mn for Genomic Analysis 

To obtain enrichment cultures, sample sediments from the Fe-Mn layers (St6Grf4_23-24_FeMn_L and St7Grf6_20-21_FeMn_L) were put in laboratory bottles (1 L) with a culture medium. This medium contained sterile Baikal water; the remaining oxygen was replaced by flushing N_2_ in the bottle. The samples were stored at 7 °C in the refrigerator. After eight months of cultivation, DNA was extracted by enzymatic lysis, followed by phenol-chloroform extraction. Shotgun sequencing was performed using intact sediments from the layer enriched in Fe and Mn (St6Grf4_23-24 and St7Grf6_20-21), and enrichment cultures from the same sedimentary layers. The qualitative results were obtained only by sequencing the DNA of the enrichment culture St7Grf6_20-21_FeMn_L (Enrichment culture S5). The failure could result from the concentrations of metals in the intact sedimentary layer, which can chelate negatively charged molecules such as DNA [59] and inhibit the polymerase chain reaction. 

### 2.7. Shotgun Sequencing, Assembly, Binning, and Annotation

Metagenomic sequencing was performed at the Novogene (Tianjin, China) using the Illumina 2 × 150 PE protocols on an Illumina NovaSeq 6000 platform, which provided 40.7 million adapter-trimmed quality-checked reads (average 59.26% of GC). De novo assembly was carried out using MEGAHIT v1.2.9 [60]. Contig statistics were conducted using QUAST [61]. Annotation of contigs was assessed with SqueezeMeta v1.6.0 [62]. Within the SqueezeMeta pipeline, RNAs were predicted using the Barrnap [63]. Open reading frames (ORFs) were consequentially obtained using Prodigal with the meta option selected [64] and then with Diamond BlastX [65] on parts of the contigs where no ORFs were predicted using Prodigal. The detected ORFs were functionally annotated against the Kyoto encyclopaedia of genes and genomes (KEGG [66]) and clusters of orthologous groups of proteins (COG [67]) databases using DIAMOND. HMM homology searches against the Pfam database [68] were performed by HMMER3 [69]. Taxonomic classification of the ORFs was undertaken using DIAMOND against NCBI’s nr database. Read mapping against contigs was performed using Bowtie2 [70]. Contigs (>1.5 kb) were binned using CONCOCT [71], MaxBin2 [72], and MetaBAT2 [73]. Combination and refining of binning results were carried out using DAS tool [74]. The degree of completeness and contamination of the resulting bins were estimated using CheckM v1.2.2 [75].

Taxonomic classification of recovered MAGs (completeness > 50% and contamination < 10%) was performed using the GTDB-Tk v2.2.6 tool [76] and the Genome Taxonomy Database (GTDB) release R214 [77]. Specific phylogenetic trees were visualized using the online iTOL tool [78]. 

To compare the 16S rRNA read classifications between metagenomic and metabarcoding data, 16S rRNA fragments among the Illumina reads (unassembled) were identified using ssu-align [79]. Thereafter, 16S rRNA fragments were aligned and taxonomically classified against the SILVA 138_1 database. For the identification of iron metabolism genes in the genomes of microorganisms from Lake Baikal, the FeGenie database was used [80].

Raw metagenomics dataset and metagenome assembled genome derived from this study are publicly available under the NCBI Bioproject PRJNA875570 and ID SAMN35796698.

## 3. Results

### 3.1. Lithology 

The lithology of the investigated sediments was typical of this basin [9]. The oxidized zone identified by the brown color of the sediments reached 23 cm in the St6Grf4 core and 21 cm in St7Grf6. Reduced interlayers and fragments of the oxidized crust, up to 1 cm thick, were more obvious in the latter core (St7Grf6). The oxidized sediments of St6Grf4 were light brown diatomaceous aleuropelitic mud. There was a dark Fe-Mn layer inside the oxidized layer at a depth of 12–13 cm. The densest Fe-Mn layer was located in the reducing part of the sediments (23–24 cm). At a depth of 24 cm and below, the sediments contained grey clay with greenish-grey interlayers. Oxidized sediments of St7Grf6 (up to a depth of 21 cm) were also light brown diatomaceous aleuropelitic mud. A dark Fe-Mn layer was found inside the oxidized layer at a depth of 6–7 cm, and the second denser Fe-Mn layer was located in the reducing part of the sediments at a depth of 20–21 cm. Below, the sediments also contained grey reduced clays. 

### 3.2. Chemical Composition of Pore Water

**Figure 2:**
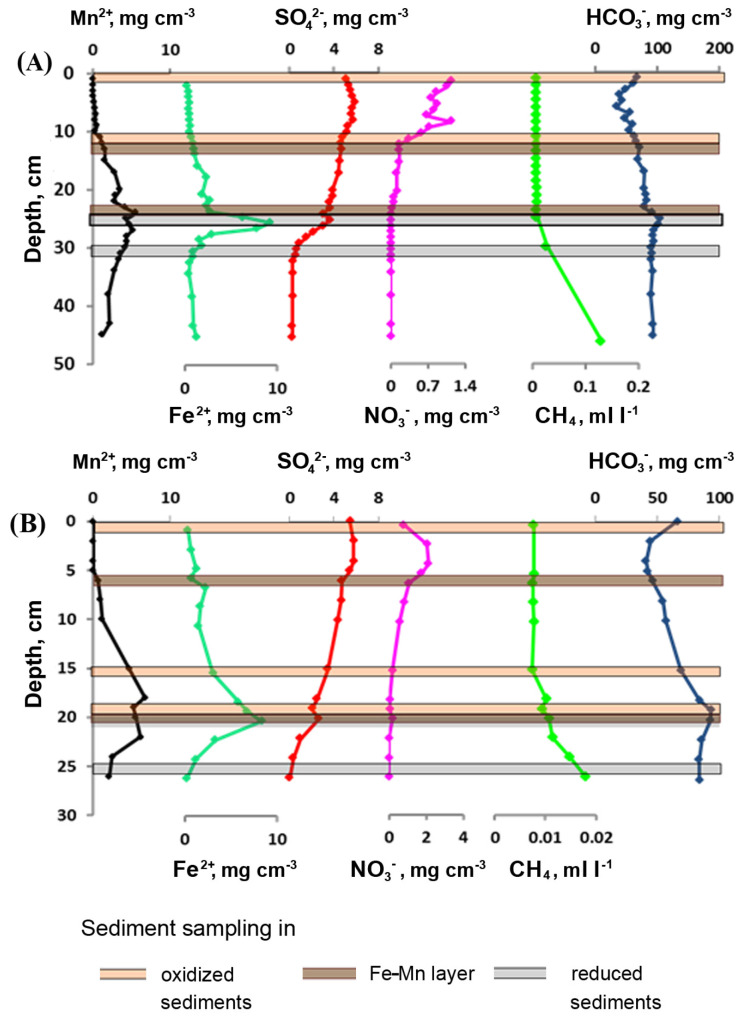
Pore water concentration profiles in the sediments of two sites in the northern basin of Lake Baikal (St6Grf4 (**A**) and St7Grf6 (**B**)).

The Eh values in the oxidized sediments of St6Grf4 varied from +233 to +272 mV, pH—from 6.7 to 7; in the reduced sediments, Eh = −82 mV; pH = 7.3. Oxygen concentration in the bottom water (the depth of water column 925 m) was 11.6 mg/dm^3^. The nitrate concentrations gradually decreased and completely disappeared at a depth of 12 cm (Figure 2); the concentrations of bicarbonate ions decreased in the first six centimeters of sediments, increasing towards the boundary of oxidized and reduced sediments as well as in the lower centimeters (30 cm). Sulfate concentrations increased in the first six centimeters, decreasing below, while the concentrations of Fe^2+^ and Mn^2+^ reached the maximum values at the boundary of the oxidized and reduced sediments and were consistent with the previous data [8]. Methane concentrations increased with different gradients from the surface to the end of the core (30 cm). In the sediments of St7Grf6, the oxygen concentration in the bottom water (900 m depth) was 11.6 mg/dm^3^; the Eh values in the oxidized layer ranged from +215 to +240 mV; pH = 7.0–7.6. The concentration profiles of the ions were, with slight deviations, similar to those of St6Grf4 (Figure 2). 

### 3.3. Alfa Diversity

The alpha diversity indices showed high bacterial and lower archaeal diversity in all sediment samples. An analysis of alpha diversity revealed that the highest indices among all sedimentary layers, which reflect latent richness (ACE and Chao1), as well as richness and evenness (Shannon and Inverse Simpson) that were characteristic of the bacterial communities from St7Grf6 at depths of 0–5 and 6–7 cm, and from St6Grf4 in the 10–11 cm layer (Table S1). The Shannon values and Inverse Simpson index varied more significantly in the St7Grf6 core (Table S1); species diversity was the most even in the St6Grf4 core. 

### 3.4. Beta Diversity

**Figure 3:**
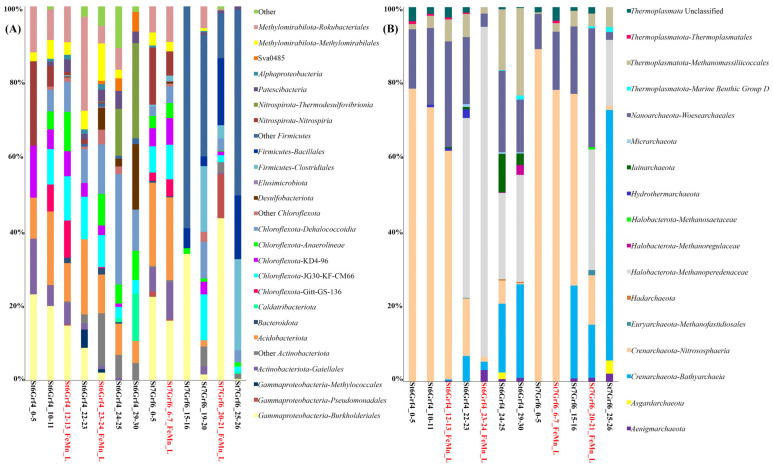
Taxonomic composition (**A**) of the bacterial and (**B**) archaeal communities in the sediments of the northern basin of Lake Baikal; more than 97% of reads. The libraries were processed using DADA2 v1.26.

Overall, 2,198,811 16S rRNA gene fragments were identified to characterize the composition of microbial communities. Bacterial communities differed both in the composition of dominant taxa and their ratio (Figure 3A). In St6Grf4, the profiles of the major bacterial communities were similar for the layers of 5–6, 10–11, and 22–23 cm, and slightly changed with the core depth, mainly in the ratios of the major phyla. Organoheterotrophic members of *Chloroflexota* (12–36%) made a large contribution to the communities of all samples. Within this phylum, the sequences of uncultured members of the family *Dehalococcoidia* and uncultured *Chloroflexota* of different clusters (KD4-96, JG30-KF-CM66, and Gitt-GS-136) predominated. We also identified heterotrophic *Actinobacteriota* (4.5–14%) that were mainly represented by the sequences of non-spore-forming anaerobic or facultative anaerobic *Coriobacteriia* and *Thermoleophilia*. 

The sequences of *Acidobacteriota* (5.2–15%) of two classes, *Acidobacteriae* and *Holophagae*, largely contributed to the communities of all samples. At the same time, the sequences of *Gammaproteobacteria* (9.8–31%) mostly contributed to the St6Grf4 communities to the occurrence depth of the Fe-Mn layer (23–24 cm). Most of the proteobacterial sequences were affiliated with the taxa represented by aerobic, microaerobic, and facultative anaerobic organotrophs of the orders *Burkholderiales* and *Pseudomonadales*, and by aerobic methanotrophs such as *Methylococcales*. Except for the community from the 29–30 cm depth of St6Grf4, within the phylum *Methylomirabilota*, other samples had anaerobic methanotrophs (order *Methanomirabilales*) oxidizing methane with the reduction of NO_2_^−^ to N_2_. The sequences of this taxon belonged to the orders *Methylomirabilales* and *Rokubacteriales* and accounted for 7 to 20% of all sequences. In two surface layers, we recorded a significant percentage of sequences of nitrite-oxidizing bacteria, *Nitrospirales* of the phylum *Nitrospirota* (4 to 20%), and in two lower layers of the St6Grf4 core—the class *Thermodesulfovibrionia* (11 to 22%). The sequences of sulfate-reducing bacteria of the class *Desulfuromonadia* (*Desulfobacteriota*) were at a maximum in the community from the 29–30 cm layer (up to 15%). Members of *Alphaproteobacteria* and *Planctomycetota* had a limited distribution, with their proportions in the communities at depths of 10–11 cm and 23–24 cm (Fe-Mn layer), amounting to 3.5–6.8% and 1.5–14%, respectively. Members of *Ca*. Curtissbacteria (*Patescibacteria*) showed a substantial percentage (up to 20.9%) in the reduced sedimentary layers (10–11 cm and below) and JS1 was at a depth of 29–30 cm (11%). 

The profiles of bacterial communities were more variable at the second site (St7Grf6, Figure 3A). Only the surface samples (0–5 and 6–7 cm) were similar between each other. In the communities of these sedimentary layers, we detected members of the phyla Nitrospirota (3–9%), *Planctomycetota* (1.6–3%), *Patescibacteria* (1–2.5%), and *Desulfobacteriota* (1%) dominated by *Methylomirabilota* (8–10.59%), *Chloroflexota* (7–27%), *Acidobacteriota* (18–19%), *Actinobacteriota* (6–9%), and *Gammaproteobacteria* (17–26%). There were significant changes in the community structure at a depth of 15–16 cm, including the Fe-Mn layers (6–7 and 20–21 cm) in the zone with the increased generation of bicarbonate ions, Fe^2+^ and Mn^2+^, as well as with methane consumption. The community mainly consisted of *Gammaproteobacteria* (29.5%) and *Firmicutes* (88%) belonging to two orders (*Clostridiales* and *Bacillales*). The latter had an insignificant percentage or were not detected in other communities of the St7Grf6 core. Members of *Chloroflexota* (1.3–17%) and *Gammaproteobacteria* (mainly order *Burkholderiales*) also dominated the community of the Fe-Mn layer at the 20–21 cm depth (48%), accounting for 1% in other communities. The contribution of *Methylomirabilota* in the communities below 15 cm was insignificant (0–3.8%).

Four dominant phyla represented archaea (Figure 3B): *Crenarchaeota*, *Halobacterota*, *Nanoarchaeota*, and *Thermoplasmatota*. Additionally, members of *Aenigmarchaeota*, *Asgardarchaeota*, *Euryarchaeota*, *Hadarchaeota*, *Hydrothermarchaeota*, *Iainarchaeota*, and *Micrarchaeota* were minor. Unlike bacterial communities, the profiles of archaeal communities were more similar to each other in the samples of two cores (Figure 3B). Members of three phyla predominated in the surface and subsurface sedimentary layers: *Crenarchaeota* (22–88.7%), *Nanoarchaeota* (2–31%) and *Thermoplasmatota* (1.8–28%). Among *Crenarchaeota* dominated aerobic ammonia-oxidizing of the family *Nitrosopumilaceae* (27–77%) and unclassified *Nitrososphaeria* (5.5–49%). A phylum *Nanoarchaeota* presented sequences of order *Woesearchaeales* (0–33%). *Methanosarcinia* (*Halobacterota*), among which anaerobic methanotrophs, *Methanoperedenaceae* (formerly ANME-2d), comprised 18 to 88%, were substantial in the underlying communities (below 20 cm). The contribution of methanogenic *Methanoregulaceae* (*Halobacterota*) was lower (to 5.8%) in the 29–30 cm layer of St6Grf4. *Bathyarchaeia* were represented *Crenarchaeota*, with contribution ranging from 11.5 to 53.5%. The communities of the buried Fe-Mn layers had a high proportion (up to 88% in St6Grf4 and up to 32% in St7Grf6) of denitrifying anaerobic methane oxidizers of the family *Methanoperedenaceae*.

**Figure 4:**
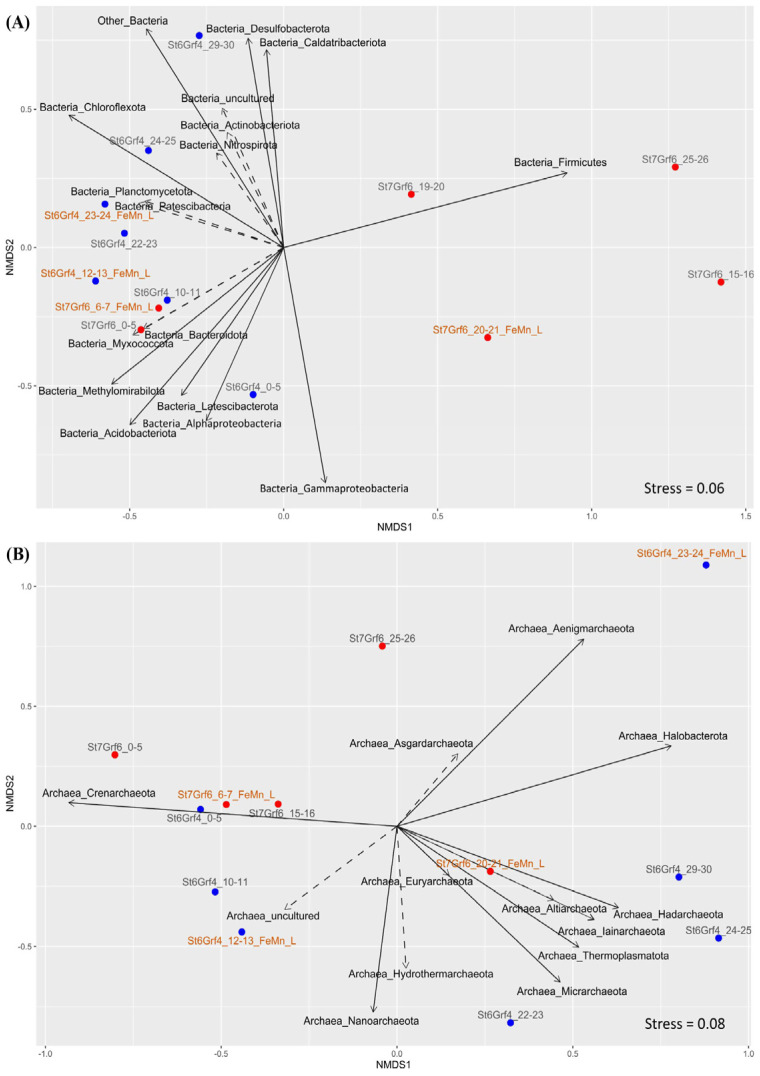
NMDS of microbial community composition of all samples (phylum level based on 16S rRNA) overlaid with relative abundance of key taxa in different sediment samples of the core. Continuous arrows show that taxa reliably correlated (*p* value < 0.05) with bacterial (**A**) and archaeal (**B**) community structure ordination. Relative distances among the samples were computed based on Bray–Curtis dissimilarities.

NMDS analysis of ASVs confirmed the difference in the structure of bacterial and archaeal communities between the cores by depth of sediments. Several clusters formed bacterial communities, and two of them (19–20 and 20–21 cm of St7Grf6) dominated by *Firmicutes* were located separately (Figure 4A). The similarity of the structure of communities in the surface and subsurface sediments of both cores and the differences below the oxygen penetration boundary was obvious (Figure 4A,B). 

### 3.5. Genomic Research

Sequencing DNA of the Enrichment culture S5 provided sequences of contigs with a total length of 824,420,296 bp. Overall, 37 MAGs were assembled with a completeness > 50% and <10.5% contamination. Taxonomic analysis of the genomes using reference sequences in Genome Taxonomy Database based on the search of the closest homologues of 16S rRNA in the NCBI database indicated the phylogenetically diverse bacterial and archaeal phyla. Among the taxa that could not be assembled above this threshold, there were some members of *Nitrospirota*, *Acidobacteriota*, *Methylomirabilota*, *Bacteroidota*, *Desulfobacteriota*, *Planctomycetota*, and *Crenarchaeota*. Eleven genomes with the assembly completeness > 50% from the described taxa were assigned to unclassified members of *Actinobacteriota*, *Gammaproteobacteria*, *Desulfobacteriota*, *Bacteroidota*, *Methylomirabilota*, *Patescibacteria*, and *Deinococcota* found in different lakes, soils, biofilms, and subsurface terrestrial fluids. Table S2 shows the characteristics of the assembled genomes, including the representation of estimated genome size (Mb) in comparison with the guanine–cytosine content (GC content) of all our MAGs. Average Nucleotide Identity (ANI) values for the bulk of the assembled genomes were below 95–96%, which is much lower than the accepted ANI species boundary [81].

**Figure 5:**
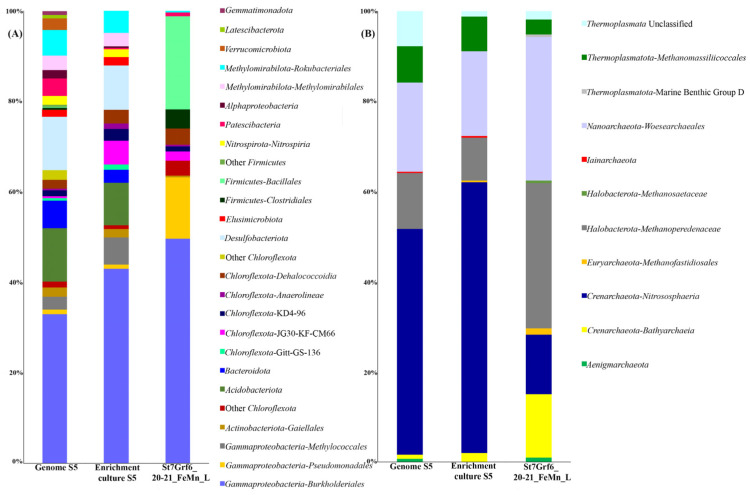
Microbial community structure (bacterial (**A**) and archaeal (**B**) communities) based on 16S rRNA gene fragments from unassembled Lake Baikal metagenomes from Enrichment culture S5 (Genome S5) compared to the enrichment culture (amplicon sequencing) (Enrichment culture S5) and 16S rRNA gene reads from a sediment sample (St7Grf6_20-21_FeMn_L).

We compared 16S rRNA gene fragments from the unassembled reads of the enrichment culture with those obtained from the analysis of amplicons of this gene in the intact sedimentary layer and the Enrichment culture S5. As shown in Figure 5, the contribution of the major phyla in the enrichment culture and the unassembled reads is similar. These samples had more identified taxa compared to the intact sample and different contribution of the same taxa. For example, an analysis of reads revealed the 16S rRNA gene sequences of *Acidobacteriota*, *Desulfobacteriota*, *Nitrospirota*, *Methylomirabilota*, and others that were absent in the library of this gene from the intact sediment. 

Noteworthily, the assembled MAGs belonged to the taxa most represented in the 16S rRNA gene libraries (Figure 5). Six MAGs represented members of *Gammaproteobacteria* (Table S2), and five MAGs represented members of the phylum *Desulfobacteriota*. Among *Methylomirabilota*, three MAGs were assigned to the anaerobic methanotrophs using nitrite as electron acceptor, while the other two were assigned to *Ca*. Rokubacteria (m8 and c9) and MAG-c71 were unclassified. Two MAGs (c8 and c11) were assigned to *Actinobacteriota*. Two MAGs also belonged to bacteria of the phylum *Planctomycetota* (m12 and c67), *Acidobacteriota* (m10 and m40), and *Bacteroidota* (m24 and c69). We obtained one MAG for the phyla *Deinococcota* (m54), *Patescibacteria* (c49), *Chloroflexota* (c76), and *Elusimicrobiota* (c55) each. Three MAGs belonged to the archaea of different phyla: *Thermoplasmatota*, *Crenarchaeota*, and *Halobacterota* (Table S2). 

### 3.6. Phylogenomic Analysis

**Figure 6:**
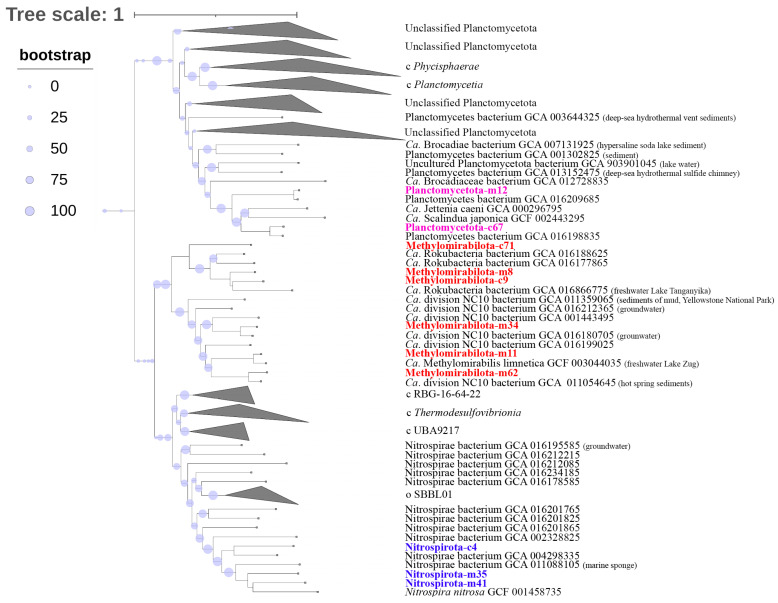
Phylogenomic tree of MAGs *Methylomirabiota*, *Nitrospirota*, and *Planctomycetota* from Enrichment culture S5 reconstructed genome. Taxonomy is shown according to the GTDB.

We constructed a phylogenetic tree based on 120 sequences of conservative marker genes to determine the phylogenetic position of bacteria of the phyla *Planctomycetota*, *Methylomirabilota*, and *Nitrospirota* as the main participants of the methane and nitrogen cycles. As shown in Figure 6, Planctomycetota-m12 and Planctomycetota-c67 clustered with the members of ANAMMOX bacteria. Three MAGs of the phylum *Methylomirabilota* (Figure 6) belonged to the order *Rokubacteriales*, and the *Methylomirabilota*-c7 genome formed an individual lineage in this clade of the phylogenetic tree. The other three genomes were phylogenetically close to bacteria of the order *Methylomirabilales*. The two of three genomes of the phylum *Nitrospirota* (Figure 6) were closely related to the species *Nitrospira nitrosa* (Comammox bacteria). 

**Figure 7:**
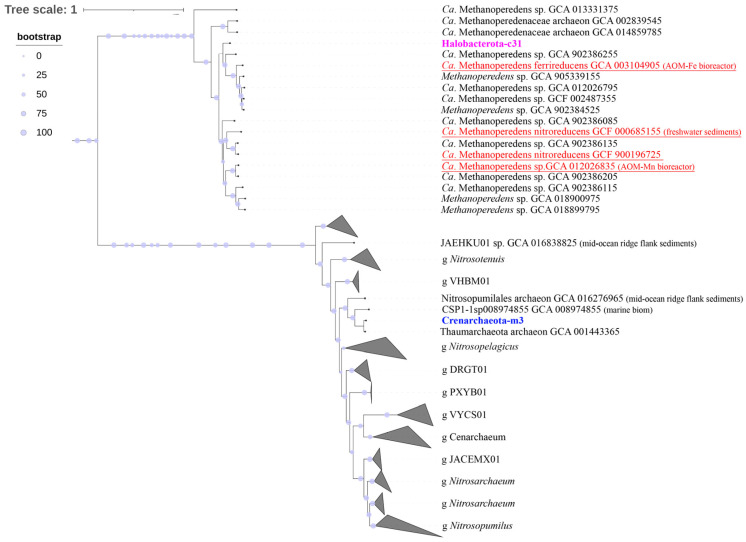
Phylogenomic tree of MAGs *Ca*. Methanoperedens (*Halobacterota*-c31) and *Nitrososphaeria* (*Crenarchaeota*-m3) from Enrichment culture S5 reconstructed genome. Taxonomy is shown according to the GTDB.

Phylogenomic analysis of MAGs of the *Halobacterota*-c31 and Crenarhaeota-m3 archaea (based on 53 conservative archaeal marker gene sequences) revealed that MAG-c31 was phylogenetically different from *Ca*. Methanoperedens nitroreducens (Figure 7). It was part of the cluster with *Ca*. Methanoperedens ferrireducens were involved in AOM with the Fe(III) reduction, forming a separate branch within this clade (Figure 7). The Crenarhaeota-m3 MAG was clustered with ammonia-oxidizing archaea of the order *Nitrosopumilales*.

### 3.7. Key Metabolic Pathways of MAGs

**Figure 8:**
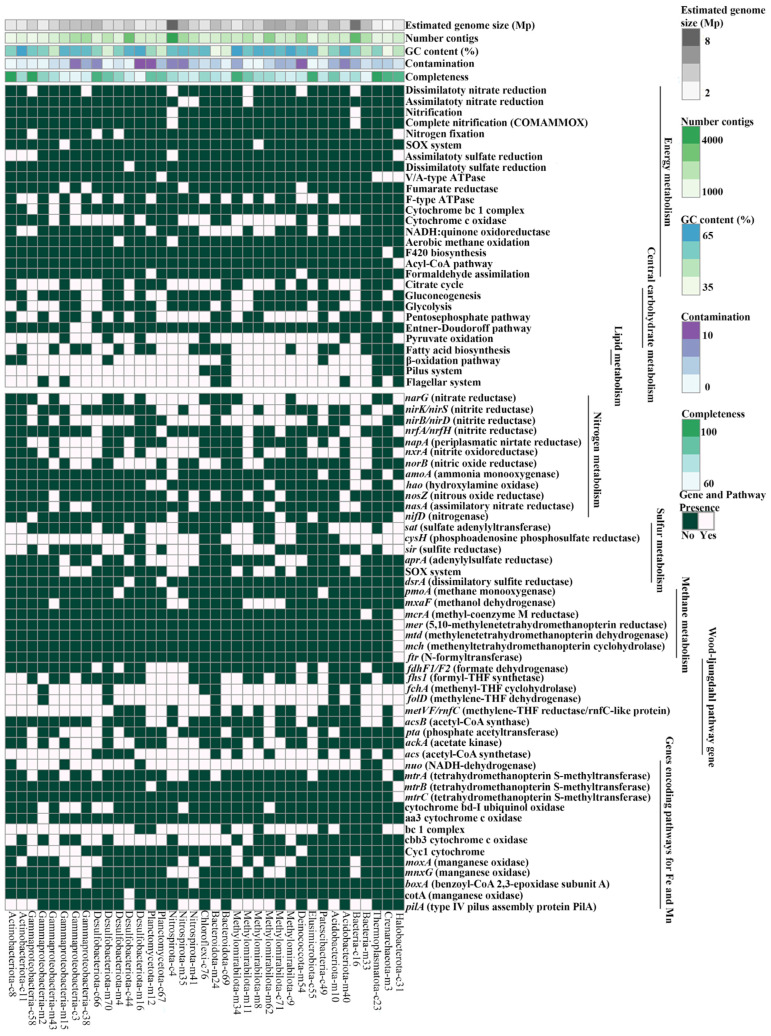
Summary of different metabolic pathways found in the 37 MAGs from enrichment culture S5. The incompleteness of MAGs has to be considered when assessing the absence/incompleteness of metabolic pathways.

The analysis of metabolic pathways in the MAGs indicated a wide range of genes that provide the participation of microorganisms in the cycles of carbon, nitrogen, and sulfur, as well as carbohydrate and lipid metabolisms (Figure 8). It should be taken into account that all metabolic pathways shown in Figure 8 were reliably detected in various MAGs, but they also can be incomplete due to their incomplete assemblage. In the analyzed genomes, the most common pathways of central metabolism are the pentose phosphate pathway (PPP; in 20 MAGs), TCA cycle (22 MAGs), and Pyruvate oxidation (29 MAGs). We identified autotrophic metabolism in ten MAGs with complete glycolysis pathways, including the Embden–Meyerhof–Parnass pathway in four MAGs (EMP; MAGs-c55; m34; m24; c16) and Entner–Doudoroff in two MAGs (ED; MAG-c3 and MAG-c38). MAG-c38 (*Gammaproteobacteria*) had all genes encoding complete PPP and ED, and MAG-c55 (*Elusimicrobiota*) had PPP and EMP. Ten of the obtained MAGs had the capacity for gluconeogenesis. To conserve energy, many (especially facultative) autotrophs use organic matter glucose production through glucogenesis. Also, most Baikal MAGs had genes of fatty acid metabolism, including synthesis and oxidation (β-oxidation) that can be used as substrates for the growth of microorganisms. The bulk of the assembled genomes had some of the Wood–Ljungdahl pathway genes. The oxidation of acetyl-CoA with the formation of acetate and generation of ATP can occur in the two-stage reaction using phosphate acetyltransferase (*pta*) and acetate kinase (*ackA*), forming acetyl-CoA with acetyl-CoA synthetase (*acs*), which is characteristic of MAGs of *Elusimicrobiota*-c55, *Nitrospirota*-c4, and some *Gammaproteobacteria*.

The genes for the complete methane cycle were present only in singular MAGs (Figure 8), and genes providing anaerobic methanotrophy and methanogenesis were more common. The key gene (*pmoA*) for aerobic oxidation of methane was present in Nitrospirota-c4 MAG, Patescibacteria-c49, and Desulfobacteriota-m4 MAGs. Gene *mxaF* in the MAGs of *Methylomirabilota* (c71, m8, m11, and m62), *Nitrospirota* (c4 and m35), and *Gammaproteobacteria* (m43) provided oxidation of methanol to formaldehyde. Genes for anaerobic methanotrophy and methanogenesis were detected in the Halobacterota-c31 MAG. The genes (*mcrA*, *mtr*, *mer*, *mtd*, *mch*, and *ftr*) encoding the central methanogenesis pathway that were responsible for the first to sixth steps of these processes were present in this genome, which implies its ability to oxidize methane through the reverse pathway. 

We identified the complete set of genes required for dissimilatory and assimilatory nitrate reduction in nine bacterial genomes assigned to *Gammaproteobacteria*, *Nitrospirota*, *Methylomirabilota*, and *Deinococcota*, as well as to one unclassified MAG (Bacteria-c16) (Figure 8). Some MAGs from Lake Baikal had denitrification genes: NO-forming nitrite reductase NirK/S, nitric-oxide reductase (Nor), and nitrous oxide reductase (Nos). The Halobacterota-c31 MAG had the *nifD* genes encoding atmospheric nitrogen fixation enzymes, Nar and Nxr nitrate reductase genes, catalyzing the reduction of nitrate to nitrite and vice versa. The presence of different genes for nitrogen metabolism in MAG of *Ca*. Methanoperedens sp. can bind methane oxidation to nitrate reduction using the *narG* bacterial enzyme. The complete pathway of ammonia oxidation to molecular nitrogen and the presence of ammonia monooxygenase (*amoA*) was identified for the Bacteria-c16 MAG, and the *amoA* gene was also characteristic of the Crearchaeota-m3 MAG belonging to the class *Nitrososphaeria* (formerly *Thaumarchaeota*). 

Genes encoding complete assimilatory sulfate reduction enzymes were identified only in the MAGs of *Ca*. Methanoperedens sp. (c31), *Actinobacteriota* (c8 и c11), Gammaproteobacteria-c58, and Nitrospirota-c4. There was the complete cycle of dissimilatory sulfate reduction in the *Desulfobacteriota*-c44 MAG, and the *dsrA* gene was also in the *Planctomycetota*-c67 MAG, while individual genes for assimilatory sulfate reduction were present in most MAGs of *Acidobacteriota*, *Methylomirabilota*, *Nitrospirota*, *Planctomycetota*, *Desulfobacteriota*, and *Deinococcota*. The complete complex of the SOX genes involved in the oxidation of thiosulfate to sulfate were identified in the MAGs of *Ca*. Rokubacteria (m8) and *Betaproteobacteria* (m15), and some genes of the SOX complex in members of *Methylomirabilota* (c9, m62, m8, and m34), *Nitrospirota* (m35 and c4), *Gammaproteobacteria* (c38, c3, and m15), and Bacteria-c16. 

Notably, the bulk of the assembled bacterial genomes had type IV pili proteins, ensuring the motility of bacteria. Among the multiheme cytochromes c (Cyc1 and Cyc2) we detected only periplasmic Cyc1 cytochromes for all MAGs of *Gammaproteobacteria*, Methylomirabilota-m34, and Deinococcota-m54. The bc1 complex genes were detected in the genomes of the bulk of the assembled MAGs. The electron transport chain of metal transport includes the cytochrome cbb3 oxidases that generally ensure the functioning of the iron and manganese oxidation pathways and are typical of Fe(II)-oxidizing microorganisms. These cytochromes were present in the MAGs of different filum. In the analyzed MAGs, we also identified different gene complexes that ensure the involvement of insoluble electron acceptors, including Fe(III) and Mn(IV), which are part of minerals. The *mtrA* iron reduction genes and the *mtrBC* genes involved in the iron reduction on the cell surface were present in MAGs. In the assembled MAGs, there were also Mn(II)-oxidizing multicopper oxidases (MCO). The *moxA* and *mnxG* were mainly present in MAGs of *Methylomirabilota*, *Nitrospirota* and *Gammaproteobacteria*; the *boxA* gene encoding bilirubin oxidase and belong to the MCO complex was identified in *Nitrospirota*-m41 and Gammaproteobacteria-c3, while CotA multiheme oxidase was only in MAGs of Desulfobacteriota-c44. 

## 4. Discussion

In terms of lithology and physicochemical characteristics, the investigated sediments of the northern basin of Lake Baikal corresponded to the previous descriptions and data [8,9]. The calculated concentration gradients and distribution profiles of major ions indicated the similarity of the processes occurring in two cores with slight variations correlating with the occurrence depth of Fe-Mn layers. As previously alluded to [9,11], dynamic growth of Fe and Mn ions occurred in the oxidized layers of the investigated cores below the maximum penetration depth of O_2_ (below 6 cm), and reducing dissolution was observed below the Fe and Mn oxidized layers, leading to the changes in the ionic concentrations of nitrate, bicarbonate, sulfate, and methane (Figure 2). According to the hypothesis of the above authors, AOM could control the dissolution of the oxidized Fe-Mn layer using SO_4_^2−^ and/or Fe(III) as electron acceptors in deeper sediments and formation of an upper dynamic Fe-Mn oxidized layer due to the diffusion flow of O_2_ from the water column into the sediments. An analysis of the diversity and structure of microbial communities in the two cores testified to the presence of bacteria and archaea with different metabolic capacities, providing various biogenesis processes, including methane formation and oxidation. The most significant changes in the community structure were between aerobic and anaerobic zones, which were also observed in five lakes of Central Switzerland and permafrost thaw ponds [82,83]. Organotrophic *Chloroflexota*, *Actionobacteriota*, and *Acidobacteriota*, as well as aerobic *Methylococcales* and anaerobic methanotrophs, *Methylomirabilota*, were predominate in the communities from the surface sediments of the cores. Noteworthy is that members of *Methylomirabiota*, as well as the species *Nitrospira*, the most identical to the bacteria from the seeps enriched in Fe, were found in the microbial communities from the sediments enriched in iron of lakes Superior [4] and Constance [6]. The archaeal diversity in this sedimentary zone of Lake Baikal differed less significantly; as in other basins, chemolithotrophic ammonia-oxidizing archaea, *Nitrososphaeria*, dominated [22,84]. Among the members of this class in the sediments from Lake Baikal, we detected archaea of the orders *Nitrosopumilales* and *Nitrososphaerales*, known participants in aerobic ammonia oxidation [85]. Ammonia-oxidizing archaea (class *Nitrososphaeria*) and nitrite-oxidizing bacteria (*Nitrospirota*) were recorded in different marine Fe-Mn deposits [26,86,87]. It was also assumed that *Nitrososphaeria* can carry out not only autotrophic metabolism, receiving energy from the ammonia oxidation, but also heterotrophic metabolism [88]. Because most of the assembled Baikal genomes had the *napA* genes, and the *narG* genes were minor, we may suggest that organotrophic nitrate reductors were mainly present in the investigated communities, and lithotrophic nitrate reductors were less present [89]. The predominance of microorganisms involved in various conversions of nitrogen sources was consistent with an increase in the nitrate concentrations in pore waters of this zone and ensured the development of bacterial and archaeal consortia participating in AOM process through nitrate- and nitrite-dependent pathways also observed in other areas of Lake Baikal [90]. In the reduced layers of both cores, we also observed the denitrifying aerobic methane oxidizers of the family *Methanoperedenaceae*, bacteria of the phylum *Methylomirabilota* and anaerobic ammonia-oxidizing bacteria of the candidate genus *Brocadiae* (*Planctomycetota*). The assembled MAGs of some *Methylomirabiota* and *Nitrospirota* had genes for oxidation (cbb3 and *cyc1* cytochromes) and reduction (*mtrA*) of Fe (II), allowing them to participate in both AOM and other metabolic processes. 

Members of the dominant *Chloroflexota* that also have functional genes responsible for the formation of the enzymes for destruction of nitrate ions could be other participants in the nitrogen cycle. Taking into account the diverse range of taxa belonging to this phylum, their active role in the formation of organic matter required for the overall functioning of communities cannot be excluded [91]. The classes Gitt-GS-136 and KD4-96, most common in soil, river sediments, freshwater lakes, numerous seabed environments [91,92], as well as in taberal sediments, including recently thawed ones [93], represented this phylum. The genomes of marine members of this phylum showed several pathways for the degradation of complex carbohydrates, allowing them to act as primary fermenters and acetogens in one microorganism without syntrophic H_2_ consumption [91]. Phylogenomic analysis of the Chloroflexota-c76 MAG from Lake Baikal indicated its identity to the *Anaerolineae* bacteria from marine sediments (Table S2), in the catabolism of which anaerobic acetogenesis plays a central role [94]. At the same time, the obtained ANI values (75.7%) of the members from Lake Baikal indicated a significant difference from its closest relative (Table S2). The genome of the Chloroflexota-c76 MAG from Lake Baikal had genes of citrate and pentose phosphate pathways, as well as some enzymes of the Wood–Ljungdal pathway, but acetate kinases were absent, which did not confirm (but does not completely exclude) a central role of anaerobic acetogenesis typical of marine members [91]. Interestingly, a decrease in the contribution of the members of the phylum *Chloroflexota* in the communities of the St7Grf6 core was accompanied by a significant increase in the proportion of the members of the phylum *Firmicutes*. A similar phenomenon was observed in clay permafrost layer from Western Spitsbergen [95] and the CRREL Permafrost Tunnel, Alaska, United States [96,97]. The authors of these manuscripts suggested that these taxa could be the markers of climate change; in particular, *Firmicutes* mark synergetic freezing of sediments (hypothesis of a marker of syngenetic sediment formation) [98]. Most likely, the unusually high contribution of *Firmicutes*, with a decrease in *Chloroflexota*, in the investigated St7Grf6 core could be due to the influence of turbidites that were common in the sediments of the northern basin of Lake Baikal and that could had been formed during the advance and retreat of glaciers in the Late Pleistocene [99,100]. The influence of ice rafting cannot also be excluded, the traces of which were often identified in the sediments of this area [101]. Our reconstructed MAGs were obtained during the cultivation of the intact community from the Fe-Mn layers under strictly anaerobic conditions in the absence of oxygen, without the addition of methane and additional sources of carbon and energy. The creation of the conditions typical of anaerobic sedimentary layers did not result in the development of *Firmicutes*; microorganisms typical of the zones with modern diagenesis developed in the enrichment culture, confirming the allochthonous origin of these bacteria. 

The sequences of *Burkholderiales (Gammaproteobacteria)*, *Gaiellales (Actinobacteriota)*, and *Clostridiales* (*Firmicutes*) that can be involved in anaerobic hydrolysis of plant polymers were also present in the communities of the investigated cores with different contribution and at different depths. As shown in [102], fermenting bacteria (*Betaproteobacteria/Rhodocyclaceae/Dechloromonas* and *Firmicutes)* and syntrophs (*Ruminococcaceae*, *Syntrophobacterales* and *Clostridiaceae*) known to decompose monomers into acetate, hydrogen, carbon dioxide, and formate, also known as precursors for the production of CH_4_, increased in the sediments of thermokarst lakes. It should also be noted that MAGs of heterotrophic *Acidobacteriota* having a significant percentage in the community of the Fe-Mn layer from the St7Grf6 core contained genes that ensure the destruction of polysaccharides, nitrate reduction, and sulfite oxidation (Sat and AprA).

Several ecological studies on geochemical data provide evidence for the capacities of AOM in combination with the reduction of Fe(III) and Mn(IV) in marine and freshwater environments [11,40,103,104,105]. Iron and manganese cycles are also known to be closely related to the carbon and nitrogen cycles [106]. In the communities of the investigated sediments from the northern basin of Lake Baikal, like in other areas of the lake [22,84], a high percentage were organotrophic *Actinobacteriota*, genomes of which also contained genes for assimilatory sulfate reduction and Fe(II) oxidation (cbb3 and bc1cytochrome oxidase). According to [107], members of this phylum can carry out chemolithotrophic Fe(II) oxidation. Members of the genus *Rhodoferax* (*Gammaproteobacteria*) may also be participants in the iron cycle. The study of bacteria of this taxon from freshwater sediments suggested that the species of this genus can not only be Fe(III) reducers but also Fe(II) oxidizers [108]. As shown in Finneran et al. [35], cultured members of *Rhodoferax* can use various organic compounds as electron donors, and Mn^4+^ oxide, fumarate, and nitrate as electron acceptors during anaerobic growth. In the MAG of *Rhodoferax* (*Gammaproteobacteria*-c38) from Lake Baikal, we identified a wide range of genes, ensuring its participance in dissimilatory nitrate reduction, conversion of nitrite ions, Mn oxidation (Mn—MoxA and MnxG oxidases), fumarate respiration (Frd), and thiosulfate oxidation (SOX system). Kuypers et al. [109] assumed that nitrate dissimilation reduction intermediates may have a more important effect on elementary biogeochemical processes such as Fe(II) oxidation in oxygen-free environments. In the MAG-c38 from Lake Baikal, there were Woods–Ljungdahl pathway genes and oxidation of acetyl-CoA with the formation of acetate and the generation of ATP, suggesting its anaerobic lifestyle. However, the absence of the Cyc2 iron oxidase in the assembled MAG-c38, which is involved in the Fe(II) oxidation, does not support this suggestion. Members of the anaerobic methanotrophic archaea, *Halobacterota*, that contributed significantly to the archaeal community of the Fe-Mn layer (Figure 3B) can be active participants of AOM processes and the reduction of Fe(III) and/or Mn(IV). Phylogenomic analysis of MAG-c31 from Lake Baikal indicated its phylogenetic identity (Figure 7) to *Ca*. Methanoperedens ferrireducens from bioreactor performing anaerobic oxidation of methane coupled to iron reduction. This microorganism is known to use multiheme cytochromes (MHC) in environments enriched in Fe-Mn to facilitate extracellular dissimilatory reduction of Fe(III) and/or Mn(IV) [40]. During AOM, different species of *Methanoperedens* sp. Showed the expression of various cytochrome *c* oxidoreductases combined with the reduction of Fe(III) oxides [40], Mn(IV) oxides [42], and nitrates [110]. In the genome of the Halobacterota-c31 MAG from Lake Baikal, we detected genes indicating its ability not to only reduce Fe(III) oxide and nitrate but also use Mn(IV) as electron acceptor during AOM. The presence of genes encoding Fe and Mn ABC transporters, as well as afuABC genes ensuring the transport of metal ions into cytoplasm, in the genomes of the bulk of taxa from Lake Baikal suggests their active role in the Fe and Mn cycle, and in AOM. Taking into account the limited number of the analyzed genes, we do not exclude the presence of other energy sources, which microorganisms inhabiting Fe-Mn layers use. 

The metagenomic analysis of reconstructed genomes of the Fe-Mn layer revealed the presence of various genes for nitrogen metabolism in most of the taxa from Lake Baikal. Genes for nitrogen fixation, nitrification, denitrification, and dissimilatory/assimilatory nitrate reduction in the reconstructed genomes were detected not only for canonical groups involved in nitrogen metabolism (ammonia-oxidizing archaea (*Nitrososphaeria*), ANAMMOX bacteria and nitrite-oxidizing bacteria of the phylum *Nitrospirota*) but are also typical of other microorganisms in the community of Fe-Mn layer. In the microbial ecosystem of Fe-Mn crusts and layers [26] a high proportion of members of *Nitrososphaeria* in communities suggests their significant role as primary producers. The oxidation of ammonia (Amo) and urea (Ure), as well as CO_2_ (3HP/4HB) fixation, were predicted as possible for the Crenarchaeota-m3 MAG from Lake Baikal [111], which indicated their chemolithoautotrophic potential. As shown in Gabello-Yeves et al. [112], genes associated with the N-cycle are more common in microorganisms inhabiting the deep area of the Baikal pelagic zone. There was also a wide range of nitrogen metabolism pathways in the investigated sample, which confirms the significant role of nitrogen compounds as energy sources, particularly for the *Methylomirabilota* bacteria involved in AOM. Analysis of the composition of microbial communities and the presence of genes for S, C, N, Fe, and Mn metabolism in the MAGs reconstructed from the Fe-Mn layer also indicated the likely coexistence of a biogeochemical cycle of various elements, creating certain conditions for the development of microbial communities that are taxonomically and functionally more diverse than those that may occur in the system of an oligotrophic lake. 

## 5. Conclusions

This is the first study that discusses the metabolic capabilities of microbial communities in diagenetic processes in the sediments of Lake Baikal containing Fe-Mn layers. Our results indicate that (i) the diversity of microbial communities significantly differs in aerobic and anaerobic zones, and between the study areas; (ii) microorganisms from oxidized Fe-Mn layers are mostly capable of autotrophic and heterotrophic carbon metabolism and less capable of methanotrophy. The bulk of the analyzed genomes revealed the capability of autotrophic diazotrophy, dissimilatory nitrate reduction, and minor genomes of denitrification. The presence of individual genes for oxidation and reduction of Fe and Mn also excludes their use by microorganisms as energy sources in the absence of other oxidizing agents. 

## Data Availability

The raw data generated from the 16S rRNA gene and metagenomes sequencing were deposited in the NCBI Sequence Read Archive (SRA) and are available via the BioProject PRJNAPRJNA875570.

## Supplementary Materials

The following supporting information can be downloaded at: https://www.mdpi.com/article/10.3390/microorganisms11071865/s1, Table S1: Number of reads, OTUs and indices of species hidden richness and evenness in the non-rarefied libraries of bacterial and archaeal 16S rRNA gene fragments (for a cluster distance of 0.03); Table S2: General features of the of the 37 Lake Baikal MAGs.
