# Supplementary material for: Microbial Communities in Ferromanganese Sediments from the Northern Basin of Lake Baikal (Russia)
Source: Microorganisms. 2023 Jul 24;11(7):1865. doi: 10.3390/microorganisms11071865 (PMC10386581; doi:10.3390/microorganisms11071865)
Supplement: Supplementary file 1 [file microorganisms-11-01865-s001.zip › microorganisms-2504233-supplementary.pdf]

Table S1. Number of reads, OTUs and indices of species hidden richness and evenness in the non-rarefied libraries of bacterial and archaeal 16S rRNA gene fragments (for a cluster distance of 0.03).

	Sediment layers, cmblf	Reads Bacteria/Archaea	$OTU_{0.03}$ Bacteria/Archaea	ACE Bacteria/Archaea	Chao1 Bacteria/Archaea	Shannon Bacteria/Archaea	Invers Simpso Bacteria/Archa
	0-5	1397/66385	454/316	808.2/356.7	738.5/373.2	5.39/2.22	96.67/4.58
	10-11	11820/49467	1022/273	1192.3/348.7	1190.3/336.7	5.72/2.11	110.83/4.31
**St6Grf4**	12-13_FeMn_L	12683/63670	981/311	1174.6/135	1175.8/359.9	5.24/2.44	33.65/5.33
	22-23	14689/34121	886/275	1081.5/355.4	1089.4/310.7	5.07/2.51	49.17/3.75
	23-24_FeMn_L	13921/33801	823/181	1032.8/306.9	1058/238.4	5.11/0.8	53.34/1.31
	24-25	13594/56584	686/257	777.4/210.8	805.2/288.3	4.99/2.91	45.90/9.54
	29-30	4104/48617	413/261	793.8/276.7	709.2/295.1	4.41/2.58	31.55/7.03
	0-5	9084/35317	917/268	1020.4/316.6	1031.9/322.5	5.77/1.76	112.98/2.82
	6-7_FeMn_L	5198/59369	813/260	1103.8/349.7	1054.8/422.2	5.65/2.04	110.58/4.04
**St7Grf6**	15-16	1525/15974	103/214	126.8/276.1	125.2/268.0	3.35/2.49	14.37/4.39
	19-20	23903/0	534/0	695.4/0	687.4/0	3.33/0	9.75/0
	20-21_FeMn_L	26328/19215	452/274	631.9/300.6	658.9/299.4	3.65/3.01	15.73/7.31
	25-26	8910/9225	318/181	414.3/213.5	419.3/216.9	3.44/2.8	10.85/5.95

Table S2. General features of the of the 37 Lake Baikal MAGs.

Phylum/class	GC Content (%)	Completeness	Contamination	ANI	Estimated genome size (Mp)	Taxonomic affiliation of MAGs (GTDB,referenced groups)	Origin of closest organism (source or reference)
Actinobacteriota-c8	55.75	100	0	82.22	2.58	Actinobacteria bacterium	Deep terrestrial subsurface fluid (GCA_003599235.1)
Actinobacteriota-c11	69.29	71.45	0.84	-	2.49	*Actinobacteriota*	N/A [1]
Gammaproteobacteria-c58	59.03	99.15	0.64	87.21	3.78	*Azonexus* sp.	GCA_016705475.1
Gammaproteobacteria-m2	56.16	75.06	0.43	-	2.23	*Burkholderiales*	N/A
Gammaproteobacteria-m43	43.55	68.94	2.08	80.03	3.6	*Methylotenera* sp.	Lake Baikal (GCA_009693255.1)
Gammaproteobacteria-m15	64.65	54.34	1.95	80.56	4.6	Betaproteobacteria bacterium	Environmental aquatic freshwater lotic sediment (GCA_001771935.1)
Gammaproteobacteria-c3	62.92	53.03	9.32	-	4.73	*Burkholderiales*	N/A
Gammaproteobacteria-c38	62.61	57.85	5.68	81.78	4.79	*Rhodoferax aquaticus*	Sao Paulo Zoo's Lake water (GCF_006974105.1)
Desulfobacteriota-c66	54.35	91.98	7.89	79.96	5.28	Desulfuromonadales bacterium	Siberian coastal permafrost (GCA_016650295.1)
Desulfobacteriota-m70	39.32	82.52	0.16	-	3.29	*Desulfobacterota*	N/A
Desulfobacteriota-m4	56.04	74.6	1.52	82.9	3.33	*Geobacter* sp.	Lake Hazen, Arctic (GCA_009886055.1)
Desulfobacteriota-c44	65.04	60.65	0.43	90.78	4.28	Deltaproteobacteria bacterium	GCA_011391555.1
Desulfobacteriota-m16	67.42	50.86	10.84	90.7	2.42	Deltaproteobacteria bacterium	GCA_011391555.1
Planctomycetota-m12	52.06	83.89	10.48	93.26	2.8	Planctomycetes bacterium (*Brocadiales*)	Rifle well FP-101 under high O_2 conditions (GCA_001828545.1)
Planctomycetota-c67	41.99	82.44	4.35	79.99	2.87	Planctomycetes bacterium	Groundwater planktonic microbiome (GCA_016198835.1)
Nitrospirota-c4	61.35	70.85	8.23	-	9.54	*Nitrospirales*	N/A
Nitrospirota-m35	63.26	63.3	8.62	91.95	5.53	Nitrospirae bacterium	Soil sample from Angelo meadow plot 2; 20cm depth; 2 days after second rain event (91mm) (GCA_001914955.1)
Nitrospirota-m41	61.62	58.78	3.45	92.78	3.63	Nitrospiraceae bacterium	Lake Baikal water (GCA_009692155.1)
Chloroflexota-c76	62.89	73.09	2.01	75.71	2.71	Anaerolineae bacterium	Marine sediments (GCA_011192015.1)
Bacteroidota-m24	35	67.24	3.45	-	4.91	*Bacteroidetes*	N/A
Bacteroidota-c69	38.91	58.8	0.51	88.65	3.88	*Sediminibacterium*	Mine wastewater (GCA_002256765.1)

Methylomirabilota-m34	69.83	93.97	5.9	81.93	2.45	Candidate division NC10 bacterium	Groundwater sites across Northern California (GCA_016180705.1)
Methylomirabilota-m11	60.01	81.27	0.16	87.39	4.08	*Ca*. Methylomirabilis limnetica	Bioreactor (GCF_003044035.1)
Methylomirabilota-m8	66.06	71.47	0	84.45	3.58	*Ca*. Rokubacteria bacterium	Groundwater planktonic microbiome (GCA_016201645.1)
Methylomirabilota-m62	65.35	71.42	2.62	84.79	5.84	Candidate division NC10 bacterium	Hydrothermal vent sediments and freshwater spring sediments (GCA_011054645.1)
Methylomirabilota-c71	62.7	62.9	5.33	-	5.87	*Methylomirabilota*	N/A
Methylomirabilota-c9	68.39	58.14	4.98	89.41	4.8	*Ca*. Rokubacteria bacterium	Sediment at 5m depth (GCA_001443385.1)
Deinococcota-m54	62.33	63.13	10.14	-	5.73	*Deinococcota*	N/A
Elusimicrobiota-c55	64.2	94.83	0	84.74	4.37	Elusimicrobia bacterium	Groundwater sites across Northern California (GCA_016182905.1)
Patescibacteria-c49	40.99	63.87	0.69	-	3.33	*Curtissbacterales*	N/A
Acidobacteriota-m10	55.32	80.37	5.16	79.32	5.53	*Chloracidobacterium* sp.	GCA_002427845.1
Acidobacteriota-m40	65.26	62.08	8.36	76.97	3.51	Acidobacteria bacterium	Soil samples from a meadow in the Angelo Coastal Range Reserve, CA (GCA_003222385.1)
Bacteria-c16	56.5	69.36	5.49	76.57	8.62	- [2]	Freshwater recirculating aquaculture system biofilter (GCF_001458735.1)
Bacteria-m33	44.2	53.61	1.88	-	4.86	-	N/A
Crenarchaeota-m3	34.6	90.93	3.44	79.02	1.54	Thaumarchaeota archaeon (*Nitrosopumilaceae*)	Sediment at 5m depth (GCA_001443365.1)
Halobacterota-c31	45.38	89.16	1.6	96.51	2.27	*Ca*. Methanoperedens sp	GCA_902386255.1
Thermoplasmatota-c23	55.38	99.07	3.5	79.93	2.07	Thermoplasmatota archaeon	Crystalline bedrock of the Fennoscandian Shield (GCA_001800815.1)

[1] N/A – not classified

[2] - – not data
